# The 15-minute city concept: The case study within a neighbourhood of Thessaloniki

**DOI:** 10.1177/0734242X241259926

**Published:** 2024-06-24

**Authors:** Maria Shoina, Irene Voukkali, Apostolos Anagnostopoulos, Iliana Papamichael, Marinos Stylianou, Antonis A Zorpas

**Affiliations:** 1 Laboratory of Chemical Engineering and Engineering Sustainability, Faculty of Pure and Applied Sciences, Open University of Cyprus, Nicosia, Cyprus; 2 Division of Transport and Project Management, Faculty of Civil Engineering, Aristotle University of Thessaloniki, Thessaloniki, Greece

**Keywords:** Urbanization, 15-minute city, SWOT analysis, GIS

## Abstract

Cities, crucial cultural hubs, mould individual and group identities. The global urban expansion, with over half the population in urban areas, presents interconnected challenges such as pollution, poverty, inequality, ageing infrastructure, resource overconsumption, land use changes, biodiversity impact and climate change. Addressing these demands ambitious actions targeting political, social and economic systems for transformative change. The theoretical framework guiding city transformation centres on an interdisciplinary approach influenced by the Smart and Green Transition. The ‘15-minute city’ concept, emphasizing human scale and urban experience, proposes that cities enable residents to meet daily needs within a short walk or bike ride. The aim of this study was the exploration of its implementation in Greek cities, particularly Thessaloniki, which reveals inherent characteristics supporting the 15-minute concept. Through an interdisciplinary approach rooted in the Smart and Green Transition framework, the research provides concrete guidance for policymakers in tailoring urban planning strategies, allocating resources effectively and crafting policies conducive to successful and sustainable urban transformations. Moreover, prioritizing public engagement highlights the significance of community involvement in shaping urban development plans, ensuring that proposed initiatives align with residents’ needs and desires. In essence, this research contributes tangible insights and actionable recommendations for Greek cities, paving the way for more liveable, resilient and sustainable urban environments.

## Introduction

The city, as an inherently human entity that is synonymous with the spatial expression of human society, encompasses a variety of human activities where urban infrastructure is characterized by various environmental alterations, shaping an adverse daily life within its boundaries (Abdelfattah et al., 2022). Cities are the places where human cultures coexist and are showcased and constitutes a social space that is fundamental to the identity of an individual or a group (Ortman et al., 2020; Perlaza Rodríguez et al., 2024). Nowadays, unprecedented urban expansion is currently taking place worldwide, with more than half of the global population residing in urban areas. According to projections from the United Nations (UN), the proportion of people living in urban areas is expected to increase to 67% by the year 2050 (Dong et al., 2024; Voukkali et al., 2023b).

As of mid-2023, more than 4.6 out of the 8 billion people worldwide live in urban areas, constituting 57% of the global population, with projections for 2030 already reaching 60%. Currently, there are 34 cities worldwide with over 10 million inhabitants. Most of these so-called megacities are in Asia (21), Latin America (6) and Africa (3). The largest city is the urban settlement of Tokyo with a population of 37.2 million residents, followed by New Delhi (32.9 million) and Shanghai (29.2 million). According to UN estimates, the number of megacities is expected to increase to 43 by 2030, with Delhi becoming the world’s largest city with an estimated population of almost 39 million (Statista, 2024). In Europe, cities occupy only 4% of its land area but host 80% of its population. This artificial land coverage increased by 3.4% in Europe between 2000 and 2006 – by far the highest proportional increase in all land use categories. As it is scattered, more than one-fourth of the EU’s territory is directly affected by urban land use. Additionally, peri-urban (discontinuous) areas developed four times faster than continuous urban areas (EEA, 2021). This expansion, often appearing in a scattered manner throughout Europe’s countryside, is referred to as ‘urban sprawl’. Cervero and Wu (1997) observed that ‘urban sprawl is difficult to define, but you know it when you see it’, often characterizing sprawl as primarily unattractive and economically inefficient settlement patterns (Vardopoulos et al., 2023). As a result, sprawl should not be considered a distinct form of settlement. Instead, it is more accurately viewed as a continuum within metropolitan areas, encompassing a spectrum from semi-compact development to fully dispersed patterns of urban growth (Cutsinger and Galster, 2006).

**Figure 1.**
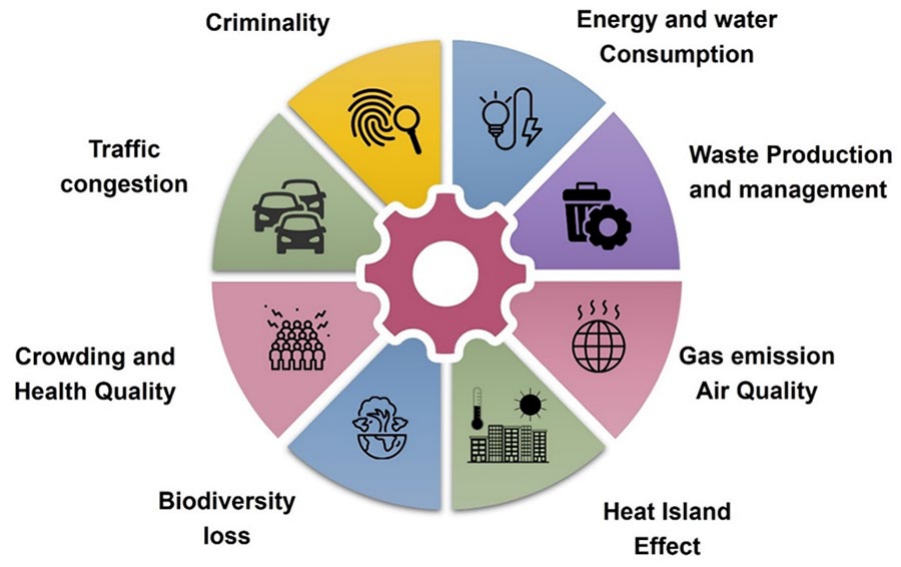
The impact of urbanization. Source: Figure created by the authors.

The intense urbanization poses an increasing threat to the sustainability of the planet. The challenges faced by the modern city involve environmental protection, the development of a green economy, circular economy, social equality, clean energy, carbon neutrality, mobility and health (Cetin et al., 2022; Liu et al., 2024b; Sui et al., 2024). Urbanization has triggered and exacerbated a series of environmental challenges, including water environment degradation, urban ecosystem deterioration, air pollution and climate change (Voukkali and Zorpas, 2021) (Figure 1). According to Chioatto et al. (2023), due to urbanization, economic expansion and alteration of living standards and demands, by 2050, annual solid waste production is expected to be 70% more than in 2016 (3.4 billion tonnes). At the same time, 47% of this accumulation will be landfilled, 22% incinerated and merely 31% recycled (Liu et al., 2019). Consequently, tackling environmental concerns in the core of urbanization has gained significant attention as a critical aspect of sustainable development and overall well-being. The notion of urban transformation is becoming increasingly prominent in both scientific and political discourse as cities undergo changes towards sustainability and resilience, aligning with the United Nations’ 2030 Sustainable Development Goals (SDGs) (United Nations, 2015). A research field around issues of urban transformations has begun to emerge, combining multiple scientific disciplines, ontologies and methodologies (Elmqvist et al., 2019; Hölscher and Frantzeskaki, 2021).

The concept of urban transformation serves as a framework that guides and shapes a more comprehensive understanding of urban change (Hölscher and Frantzeskaki, 2021). From one perspective, ‘transformation’ acts as a tool for describing and grasping the continuous, intricate and disputed processes and dynamics unfolding in cities. It helps illuminate how these dynamics reshape urban functions, address local needs and influence interactions between cities and their surrounding areas (Iwaniec et al., 2019). On the other hand, a ‘transformative’ outlook offers a prescriptive direction, emphasizing the necessity for fundamental and systemic changes to effectively address enduring social, environmental and economic issues. The goal is to deliberately move towards creating sustainable and resilient cities in the long term (Ahmad et al., 2023; Grainger-Brown et al., 2022). Crane et al. (2021), outlines four key points for implementing urban transformation: (i) must occur at a pace and scale not seen in the past, (ii) it requires ambitious, integrated actions at the city level for health and the environment, (iii) accelerating city actions necessitates changes in political, social and economic systems and (iv) Systems science, urban structures and processes are essential for promoting innovative action.

### Smart and Green Transition

Efforts to promote urban sustainability date back to the late 1980s, notably with the publication of the Brundtland report (Brundtland, 1987). Aligned with the report’s agenda, urban sustainability initiatives aim to prioritize the needs of both current and future generations by endorsing urbanization patterns and implementing plans and policies that safeguard the needs of future generations.

The design and implementation of smart green cities are rooted in the fundamental principle of sustainable development, recognizing the close connection between sustainability and smart urban initiatives (Di Marino et al., 2023a; Masuda et al., 2022; Sharifi et al., 2024). Consequently, global attention has increasingly focused on urban areas and cities as crucial players in achieving SDGs (OECD, 2022). At the same time, the SDGs correspond as a steppingstone for the European Green Deal which targets carbon neutrality by 2050, accompanied by a gradual reduction in emissions (a 55% decrease in greenhouse gas (GHG) emissions by 2030 compared to 1990 levels).

The SDGs highlight the urgent need for collective action to address challenges faced by urban areas (Visvizi and del Hoyo, 2021; Xiao et al., 2023; Xu et al., 2020). The 2030 Agenda framework requires local authorities to tackle specific challenges and priorities through SDG adaptation, allowing them to undertake activities at the local level (Jain et al., 2023). +++The perspective of sustainable urbanization presents significant opportunities to combat poverty, inequality, unemployment, climate change and other pressing global challenges across various spatial levels, from the global to the local level. Urban-related targets are prominently featured in SDGs 11, 12 and 15, followed by SDGs 6 and 3 (Akuraju et al., 2020; Allam and Dhunny, 2019; Berisha et al., 2022; Koch and Krellenberg, 2018). In contrast, there is less direct representation of the ‘social goals’, encompassing SDG 1, SDG 5 and SDG 10, which place a higher priority on social and human well-being (Blasi et al., 2022; Cai et al., 2023; Rocha et al., 2015). The integration of these goals into sustainable urbanization efforts is crucial for fostering holistic and inclusive development.

At the same time, key focal point of sustainable transition of urban settings within the framework of the SDGs is the circular economy concept. The Circular Economy Action Plan seeks to expedite the transition to a sustainable and circular economy while its primary objective lies in empowering consumer knowledge regarding product origins, sustainability and other thus establishing sustainable production and activity, which closes the loop of waste generation (European Commission, 2015). The circular economy represents an economic model that fosters competitiveness, innovation and job creation, thereby influencing the long-term resilience of cities (Papamichael et al., 2023b). The shift from a linear to a circular economy goes beyond a mere adjustment to mitigate negative impacts on physical space resulting from the inefficient management and use of resources (Music, 2019). The noteworthy R strategies of circular economy are not limited to the three strategies (reduce, reuse, recycle), but comprise of many more R (i.e. remanufacture, refurbish, rent, refuse, recover, redesign, etc.) offering innovative possibilities at various stages of a supply chain (Garcia-Saravia Ortiz-de-Montellano and van der Meer, 2022; Morseletto, 2020; Papamichael et al., 2023b, 2024). Regarding urban planning, circular economy should be in the epicentre of a sustainable city design, as the integration of circular concepts like industrial symbiosis binds strictly into sustainability (Ažman Momirski et al., 2021). Due to the escalating challenges faced by numerous cities in securing raw materials and the complex nature of industrial parks that often coexist within urbanized areas inhabited by people, the concept of industrial symbiosis emerges as one of the most effective principles within the circular economy framework, with dual benefits of positively impacting the environment and contributing to economic gains (Music, 2019).

**Figure 2.**
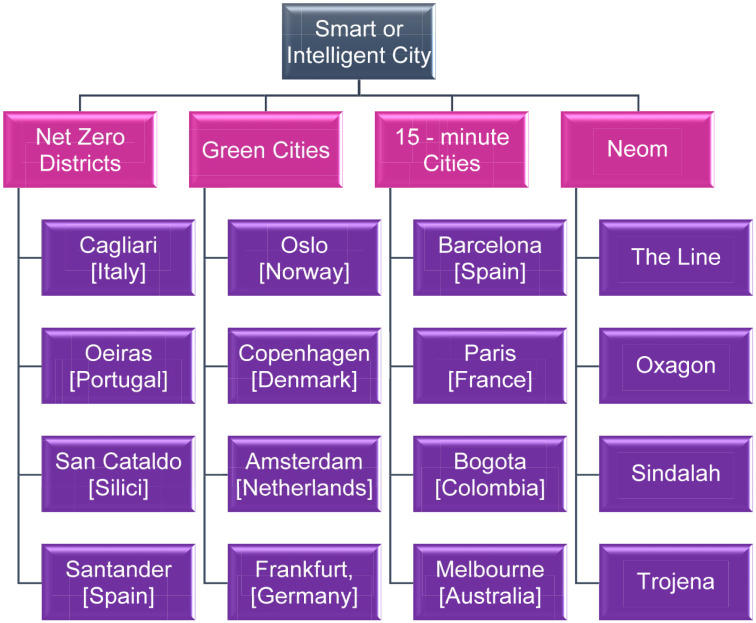
Case studies of smart or intelligent cities worldwide. Source: Figure created by the authors.

The theoretical framework guiding the continuous transformation of cities and their planning is centred around an interdisciplinary approach under the influence of ‘Smart and Green Transition’, combining contributions from urban studies, smart city systems, sustainability and climate change, spatial planning, transition management and system innovation research (Chen et al., 2024; Hui et al., 2023). The combined smart–green transition shapes 21st-century cities and encompasses all urban ecosystems, including areas of manufacturing, energy, utilities, transportation, services and housing (Ban et al., 2023; Yin et al., 2023). The digital (or smart) transition serves as the primary lever for this urban transformation, involving the implementation of smart systems, sensor networks, the Internet of Things, cloud computing, big data, artificial intelligence and other digital technologies that are transforming all urban ecosystems and planning strategies simultaneously (Franchina et al., 2021; Stamopoulos et al., 2024). The green transition is another significant lever of urban transformation with systemic impact. Guided by sustainability goals, circularity, clean energy and climate change adaptation, it broadens the horizon of urban change due to its cross-cutting nature across ecosystems, scientific fields and spatial scales (Wang, 2023; Yan et al., 2023; Zhao et al., 2023). Figure 2 presents the main models of green/smart city planning that have been adopted globally or are currently in use, enhancing their sustainability and making them more environmentally friendly for their citizens.

### 15-Minute city concept

The increasing recognition of the multidimensional nature of the concept of a ‘smart city’ has led to a shift in model from a technology-centric approach to more holistic approaches that recognize the central role of social, economic and institutional forces (Hu et al., 2023). The impacts of the smart city concept on various urban dimensions have been predominantly viewed positively, particularly in prompting transformative changes in urban infrastructures, including institutional, physical, social and economic aspects (Lara et al., 2016; Yigitcanlar et al., 2018). The cutting-edge outcomes of the smart city concept have given rise to other notions, such as the concept of the ‘15-minute city’, which is thought to leverage the foundations of the smart city concept in different ways (Di Marino et al., 2023b; Murgante et al., 2024a).

The 15-minute city stands at the opposite end of the spectrum in modern urban planning, countering the development of infrastructures that have contributed to spatial separation due to various functional specializations. The worsening division of space and time has resulted in creating opposing poles, diminishing the intrinsic value of life itself: the value of time in the urban environment (Papadopoulos et al., 2023; Zhang et al., 2023). The C40 Cities Climate Leadership Group, a network of approximately 100 mayors from around the world focusing on climate change and sustainability, has published an agenda for building more just and sustainable cities. The agenda endorses the ‘15-minute city’ as an idea to make urban areas less dependent on cars in the fight against climate change. The C40 has initiated a global initiative, and in 18 cities worldwide, it has sparked the implementation of specific projects (C40 Knowledge, 2020).

Modern urban areas, with their current structure and functioning, serve as hubs for a significant number of activities, creating the need for increased mobility to meet the daily needs of citizens (Chu et al., 2022; Law et al., 2023). Urban planning until now has largely been centred on the development of car usage, offering numerous possibilities in terms of mobility, the economy and social interactions. The continuous rise in the mobility of residents and goods, coupled with the simultaneous increase in car usage, has resulted in significant challenges in urban areas, impacting the quality of life for citizens, the environment, the economy and transportation systems (Petrović et al., 2016; Sun et al., 2023). In recent years, the policy for transportation system planning has undergone a drastic shift (Yucesan et al., 2024). The conventional approach to transportation planning, where the main priorities were minimizing travel time and increasing mobility, has been replaced by the principles of sustainable urban mobility. The primary goals now include increasing accessibility and promoting the balanced development of all modes of transportation (Davis and Altschuler, 2018; Tammaru et al., 2023).

**Figure 3.**
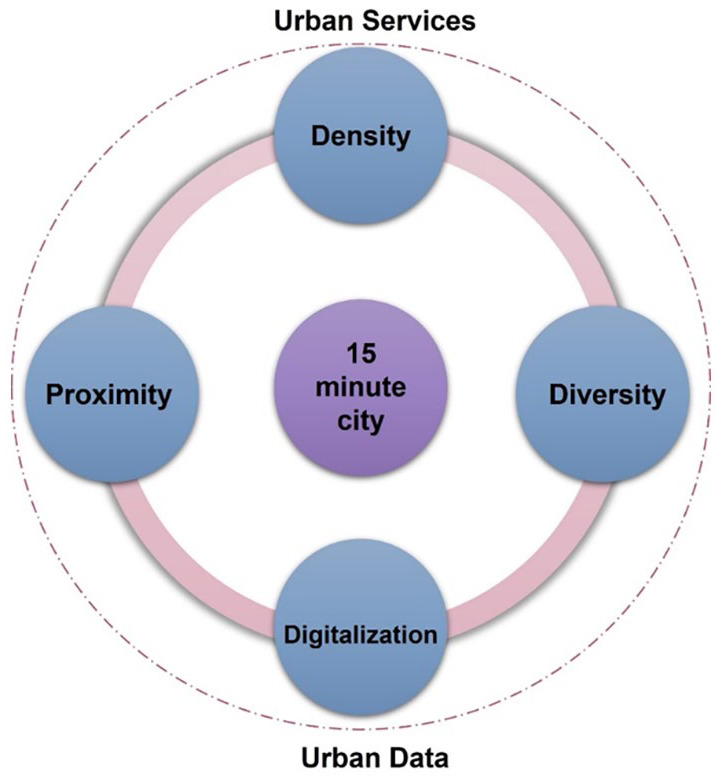
The 15-minute city framework (Moreno et al., 2021).

The model of the ‘15-minute city’ for urban development and urban planning, proposed in 2016 by Professor Carlos Moreno (Moreno, 2016), represents a relatively new way of thinking about urban planning that focuses on human scale and the experience of the city (Allam et al., 2022b; Liu et al., 2024a; Moreno, 2016; Poorthuis and Zook, 2023). Moreno’s idea, as an evolution of Banister’s model and his research on ‘polycentric city’ and ‘chrono-urbanism’ (the integration of the time dimension into urban planning, combining places, movements and time), suggests an approach to design based on ‘proximity’. Its fundamental assumption is that cities should be planned so that, within a 15-minute walking or biking distance from their residence, citizens can meet all their daily needs: work, home, food, health, education, culture, sports and recreation (Moreno, 2022). It represents a promising vision for sustainable urban living, prioritizing accessibility, ease and quality of life in a compact geographical area (Murgante et al., 2024b; Khavarian-Garmsir et al., 2023; Papas et al., 2023). According to Moreno’s design for the 15-minute city, the four key pillars of city planning are: (i) *proximity*: the essence of the 15-minute concept is to decentralize urban amenities and bring them closer to the existing built and planned quarters, rather than concentrating every citizen in areas that already have these amenities, (ii) *density*: for effective and sustainable service to the population, each area should have an optimal and balanced population size, (iii) *diversity*: the presence of mixed-use neighbourhoods is crucial for sustaining economically vibrant cities, ensuring an adequate supply of housing and promoting both sustainable and inclusive practices and (iv) *digitalization*: Utilizing various digital platforms enables the engagement of resident participation, real-time delivery and inclusivity in urban planning and management processes (Figure 3) (Moreno, 2016; Moreno et al., 2021).

Therefore, emphasis is placed on the neighbourhood as a fundamental element of spatial and functional organization (Khavarian-Garmsir et al., 2023; Pozoukidou and Chatziyiannaki, 2021). The idealistic outcome is the development of integrated, self-sufficient neighbourhoods designed to ensure citizens have safe and convenient access to their daily needs (Balletto et al., 2021; Bocca, 2021; Noworól et al., 2022). Summarizing Moreno’s views, he outlines the three fundamental characteristics of the 15-minute city are: (i) the city rhythm should align with people, not cars; (ii) each square meter of urban space should serve multiple functions and (iii) neighbourhoods should support living, working and thriving.

One of the main aspects of the 15-minute city is the reduction of private car dependency by shortening and making more convenient trips to access various amenities and activities through a denser and more diverse land use (Allam et al., 2022a). This design encourages walking and cycling due to the decreased distances and time required to reach different activities (Ek et al., 2021; Liu et al., 2020). Research indicates a positive correlation between the likelihood of people walking and cycling for transport and the diversity of land use mix and the availability of local amenities, shops and workplaces (Hatamzadeh et al., 2017; Kuss and Nicholas, 2022).The reduction in private car traffic leading to decreased air pollution, enhancing the quality of the urban environment. Considering the average emissions of current gasoline vehicles, reducing car traffic is expected to decrease CO_2_ emissions significantly. For instance, one less trip by car is estimated to reduce emissions by 67% (Quarmby et al., 2019; Woodcock et al., 2018). A scenario in a dense city environment like Paris shows that a 20–25% reduction in car traffic could lead to a 27% decrease in PM 2.5 emissions and a 38% decrease in NOx emissions (Brand et al., 2021).

The implementation of a 15-minute neighbourhood can also have economic benefits. By encouraging local businesses to establish themselves in these neighbourhoods vibrant and diverse communities can be created. The local distribution of businesses and hence workplaces proximity in such cities could contribute in the economic sustainability of the urban setting by reducing energy consumption, transport related emissions, reduction of unemployment and poverty and increase in opportunities of individual for income security (Jin and Paulsen, 2018). Additionally, residents of a 15-minute city can save on transportation expenses that car-dependent households typically incur on fuel, vehicle maintenance, parking fees and commuting (Olivari et al., 2023). The transportation cost for households in walkable districts can reach half the expenses of a car-depended area (Badawi et al., 2018). At the same time, due to the compact nature of neighbourhoods in the 15-minute city, associated infrastructure, maintenance and development costs are lower compared to areas characterized by low-density, segregation and car dependency (Khavarian-Garmsir et al., 2023).

Another important advantage of the 15-minute city is its ability to promote social cohesion and interaction within communities. By enhancing the sense of local identity and belonging, residents are more likely to engage in neighbourhood activities and support local businesses, thereby strengthening the local economy and enhancing social resilience. Recognizing the importance of social and structural arrangements in shaping the stability of smart and sustainable communities has an immense effect in participation in an energy community can be primarily influenced by familial and social networks (Biancardi et al., 2023a). Moreover, the emphasis on pedestrian and bicycle-friendly infrastructure not only encourages physical activity but also contributes to a healthier and environmentally friendly lifestyle (Middleton, 2016; Thondoo et al., 2023).

The model of the ‘15-minute city’ constitutes a challenge of the modern era. Taking into consideration that dozens of cities worldwide are already implementing appropriate policies and actions to adopt the principles of this specific urban model, this article aims to study the viability of implementing this model in a specific area or neighbourhood in the city of Thessaloniki.

While the concept itself has gained attention globally for its emphasis on human-scale urban experiences and sustainable living, its application in specific contexts, such as Greek cities, has been relatively underexplored. By investigating Thessaloniki’s inherent characteristics that support the 15-minute concept, the study offers unique insights into how this transformative urban model can be adapted and implemented in a Mediterranean urban setting. The interdisciplinary approach influenced by the Smart and Green Transition framework adds further novelty by incorporating multiple dimensions of sustainability and resilience into the analysis. Policymakers can leverage these findings to tailor urban planning strategies, allocate resources efficiently and develop informed policies aimed at achieving successful and sustainable urban transformations. Moreover, the emphasis on public engagement and collaboration underscores the importance of community involvement in shaping the future of cities, ensuring that the proposed changes align with the needs and aspirations of residents. Overall, this study contributes to the evolving discourse on urban sustainability by offering context-specific insights and actionable recommendations for Greek cities and beyond.

Using a Geographic Information System (GIS) to retrieve high-resolution spatial data, the 5th Municipal Community of Thessaloniki was investigated, at the same time, the results obtained from the GIS model were analysed using a SWOT (Strengths, Weaknesses, Opportunities, Threats) analysis (Amato et al., 2021; Ravichandran et al., 2022). SWOT was employed to provide valuable foundation, data sharing and knowledge to decision and policymakers as well as urban planners, engineers and academia, by offering strategic and targeted insights to guide an effective urban planning strategy.

## Methodology

### Definition and Characteristics of the Study Area

**Figure 4.**
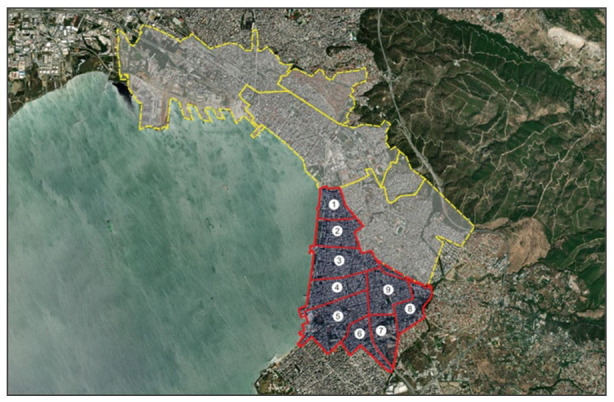
The administrative structure of the city of Thessaloniki and the examined boundaries of the neighbourhoods of the 5th Municipal Community.

The city of Thessaloniki is the second-largest municipality in Greece and a major transportation centre, as well as an economic, industrial, commercial and political centre for the southeastern Europe. According to the 2021 census (ELSTAT, 2021), the city of Thessaloniki had a population of 309,617, whereas the metropolitan population approximates 1 million inhabitants. The municipality comprises six municipal communities. The 5th Municipal Community defines the examined study area. It is the largest one and occupies 40% of the area of the municipality. It constitutes the eastern sector of the Municipality of Thessaloniki, which extends almost to the limits of the historical centre and extends along the waterfront. It is a densely built and densely populated area with few small cores of free or green spaces, mainly playgrounds, in the compact part of the community, where housing and commerce predominate, as according to the prevailing trend in construction until the mid-1990s, the ground floor of each building were intended for a shop. The situation is slightly different at the borders of the community, where green spaces, such as parks and the recently large-scale redevelopment of Thessaloniki’s waterfront enhance the quality of life of the residents. Its population of 132,351 inhabitants is distributed across nine neighbourhoods which serve as territorial units for the planning of local facilities and services of the city. Specific criteria for selecting the 5th Municipal Community as the study area were adopted in the initial phase of the study: (i) *Topography and proximity*: The selected study area has a compact urban layout, meaning a high population density that facilitates the idea’s implementation, (ii) *Transport network and infrastructure*: The study area has a well-established public transportation network and therefore has the potential to discourage private car usage by promoting the attractiveness of public transport and enhancing the mode shift in this mean of transport, (iii) *Environmental sustainability*: According to rates of pollutant emissions observed during the last decade in the selected area, it is claimed that the residents experienced high rates of air pollution level. This highlights the need for mitigation measures and to promote environmental sustainability and (iv) *Equity and inclusivity*: The selected area is characterized by trips of marginalized communities (e.g. Center for Education and Rehabilitation for the Blind). The benefits of the 15-minute approach can address issues related to inequalities. Figure 4 illustrates the administrative structure of the city of Thessaloniki. The examined study area is highlighted. The boundaries of the neighbourhoods of the 5th Municipal Community are identified as well.

### Data collection

The data collection is based on a review conducted on digital data located in reliable sources, such as the ‘Open Data Portal of the Municipality of Thessaloniki’, the ‘Geographic Portal – WebGIS of the Municipality of Thessaloniki’ and the ‘Online Geographic Information Portal of the Municipality of Thessaloniki’. Additionally, for mapping transportation infrastructure (bus and metro stops), data from reliable maps of the ‘Greek Metro S.A.’ and the ‘Thessaloniki Urban Transport Organization’ were utilized. In cases where this was deemed necessary (i.e. in cases of absence of official data from a particular geographic portal), and for the verification of the extracted data, the collection and utilization of digital data were performed through self-digitization. For this purpose, digital data from the ‘Google Earth’ software were used. Concerning essential service points such as pharmacies and supermarkets, no search and mapping were conducted, as they are numerous and located at short distances throughout the community. At the same time, temples were included since, in a country with strong customs and traditions related to religion, the visits to temples, especially on weekends, are significant for many people.

### Identification of services and locations considered for the analysis

In total, 14 types of services were selected to be considered for the analysis according to an extensive literature review of similar studies regarding the concept of the 15-minute city (Calafiore et al., 2022; Capasso Da Silva et al., 2020; Ferrer-Ortiz et al., 2022; Glock and Gerlach, 2023; Pozoukidou and Chatziyiannaki, 2021). These types of services can be described by four main categories: (i) education services, (ii) health and social services, (iii) entertainment services and (iv) public transport services.

Table 1 presents the categories and the source of the open geospatial data that were retrieved and further analysed. The dataset of the main categories is further divided into subcategories (types of services) depending on the scope of their use. The number of identified locations per subcategory is presented as well. It is noted that all extracted data were further analysed and verified to ensure reliability and validity.

**Table 1.** Types and locations of services considered in the analysis.

Type of services (locations)	Type of services (locations)
(a) *Education services*	(c) *Entertainment services*
Kindergartens^1^ (24)	Libraries^1^ (3)
Elementary School^1^ (26)	Theatres^1^ (6)
Middle and High School^1^ (19)	Cultural Institutions^1^ (11)
–	Green areas^2^ (96)
(b) *Health and social services*	Playgrounds^1^ (40)
Hospital/neighbourhood health centre^1^ (22)	(d) *Public transport services* Bus stops^3^ (127) Metro stations (future)^3^ (7)
Public and municipal services^1^ (29)	
Churches^3^ (14)	

1 Geoportal Thessaloniki (Thessaloniki Municipality, n.d.)

2 GET SDI Portal Thessaloniki (GET SDIPortal, n.d.)

3 OSM data (OpenStreetMap (OSM), n.d.).

Except from the above, spatial data regarding land uses of the examined study area were extracted and further analysed as well. Table 2 classifies the total number of land use categories that were identified in the examined study area. It is observed that the categories of residential and mixed residential occupy a significant proportion of total land use categories in the study area (almost 74%).

**Table 2.** Land use classification in the examined study area.

Category of land use (total number)	Category of land use (total number)
Sport services (16)	Special land uses (2)
Residential (672)	Central Government services (4)
Green area (96)	Medical care (8)
Industry of low/medium pollution (1)	Cultural service (25)
Mixed residential (247)	Social services (14)
Public transport services (1)	Neighbourhood centres (102)
Education (59)	Tourism/recreation purposes (1)

### Spatial analysis

The retrieved high-resolution spatial data were utilized in GIS software (http://qgis.org) to further examine the concept of the 15-minute city. Various GIS models and techniques have been utilized to analyse and identify the potential of the 15-minute city concept (Akrami et al., 2024; Ferrer-Ortiz et al., 2022; Li et al., 2019). GIS-based models are critical tools in cases of examining the conditions of accessibility to neighbourhoods services. Among their potentials, proximity visualization and quantitative analysis can enhance the methodological approach. GIS-based measurement tools can provide precise and valid outcomes by utilizing the distances depicted on the graphs (Caselli et al., 2022). Moreover, GIS can provide efficient methods that can be applied to characterizing a neighbourhoods suitability for active transport through the analysis of location-based data related to the topographical, cadastral, infrastructural and architectural features of urban areas (Gorrini et al., 2023).

Therefore, the current research work was based on an extended spatial analysis carried out through the application of GIS. The GIS analysis was based on a series of location-based data retrieved from different open-source repositories, as presented above. It is noted that the ‘GGRS87/Greek Grid’ geographic reference was selected for the analysis.

**Figure 5.**
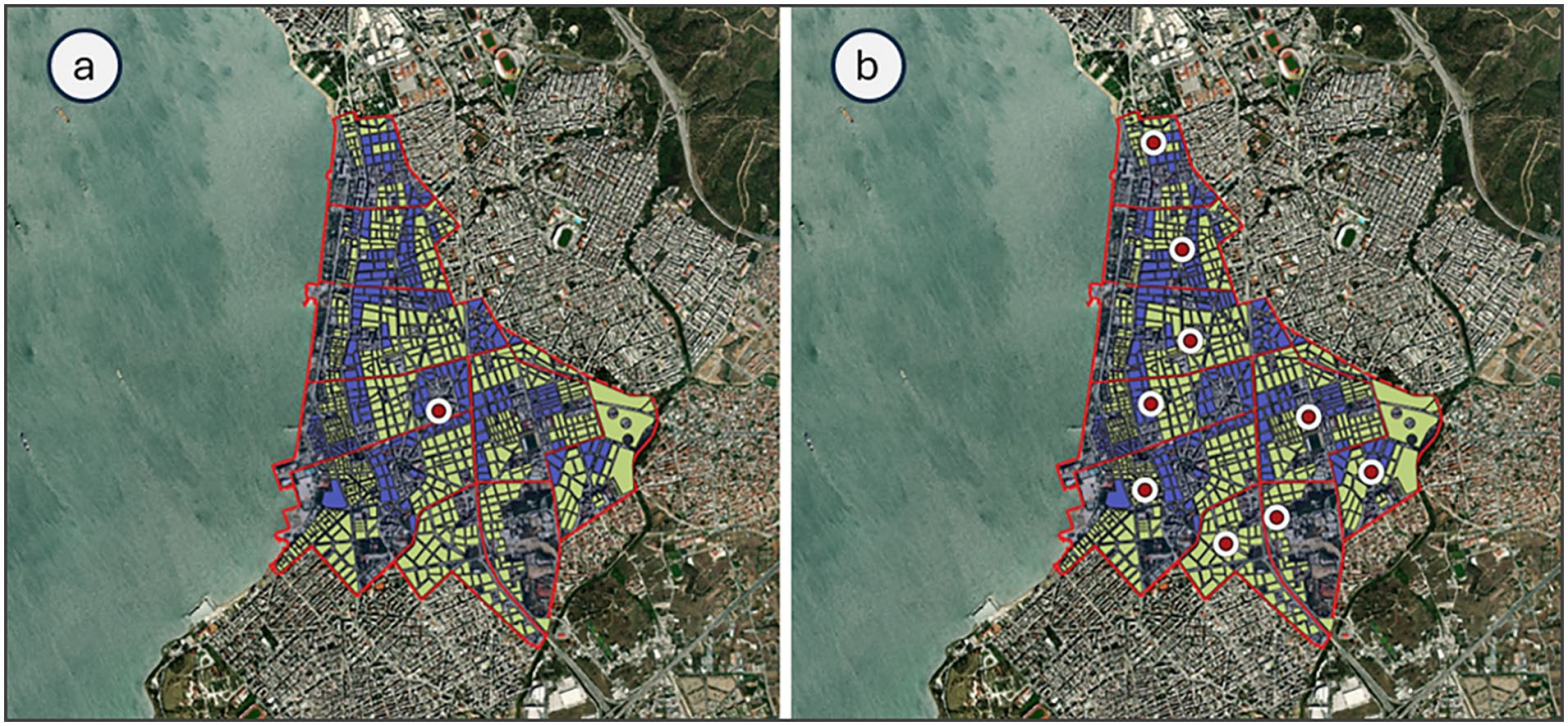
Resident centroids (a) for the examined study area, and (b) per each neighbourhood.

To examine the accessibility of the 5th Municipal Community of the city of Thessaloniki, the centroid, which is also known as the geometric centre of an area, was initially calculated for both residential and mixed residential land uses and identified in the examined study area as illustrated in Figure 5(a). It should be mentioned that centroids are points that represent all residents’ trip productions and attractions for a defined area. However, as the territory of the study area can meaningfully be distinguished on a micro-level structure, it was considered sufficient to carry out the analysis on a neighbourhood level, to better capture the level of accessibility of the residents. Therefore, for each neighbourhood, the centroids of both residential and mixed residential land uses were calculated and identified as presented in Figure 5(b).

For each resident centroid, three concentric circles were identified, representing the part of the examined area that could be reached by the residents via a 5, 10 and 15 minutes both walking and cycling from the centroid, based on the Euclidean distance. The 15-minute threshold was adopted as it is the one at the base of the 15-minute city concept. The 5- and 10-minute thresholds were selected considering the context that certain services could be accessible by foot in less than 15 minutes. More specifically, 5-minute walking trips represent the accessibility to services and amenities required daily. On the other hand, 10-minute walking trips usually represent parks, schools and medical services (Staricco, 2022).

It should be noted that the choice of the Euclidean distance for the circular buffers was preferred against the road network buffers (i.e. isochrones) as the last method is more complex, requiring a higher level of computer resources. However, the usefulness of circular buffers can dramatically be limited in case the topology of the examined area is characterized by physical restrictions and social structures, such as natural features (e.g. rivers or lakes) (Crosby et al., 2018; Oliver et al., 2007). Nevertheless, as the topology and the road network of the examined study area is not characterized by significant restrictions and limitations (i.e. significant variations for speed limits or disproportion between one-way and two-way streets), circular buffer implementation was presumed to be an effective method.

### SWOT analysis

SWOT analysis is a cognitive process that involves studying the interrelations between the internal and external environments of an organization, territory or sector. It is based on a mixed evaluation, combining subjective and objective elements, of SWOT (Amato et al., 2021; Papamichael et al., 2023a; Voukkali and Zorpas, 2021). The integration of both subjective and objective factors provides a nuanced and detailed understanding of the situation being studied. At the city level, SWOT analysis plays a vital role in assessing the municipality’s internal strengths and weaknesses alongside external opportunities and threats. By systematically examining the internal strengths and weaknesses of a city, alongside external opportunities and threats, urban planners gain valuable insights crucial for effective decision-making and sustainable development. SWOT analysis helps identify the city’s unique assets, such as infrastructure, cultural heritage or human capital, which can be leveraged to enhance liveability and competitiveness (Gkoltsiou and Mougiakou, 2021). Simultaneously, it sheds light on areas needing improvement, whether its transportation systems, affordable housing or environmental sustainability measures. Moreover, by pinpointing external opportunities like economic growth sectors or technological advancements, urban planners can align development strategies to maximize positive outcomes. SWOT analysis serves as a foundational tool for urban planners to create comprehensive, data-driven strategies that address current challenges while capitalizing on future opportunities, ultimately fostering vibrant, inclusive and resilient cities for residents and visitors alike (Heikoop et al., 2024; Voukkali and Zorpas, 2021).

A conceptual analytical model, specifically the SWOT analysis, was employed to examine the interplay between internal factors (Strengths (S) and Weaknesses (W)) and external factors (Opportunities (O) and Threats (T)) within the realm of 15-minute cities in Thessaloniki (Díaz-López et al., 2021; Papamichael et al., 2023b; Zorpas, 2020). To conduct the SWOT analysis, a team of experts has been carefully chosen based on their expertise comprising of: five experts on urban planning (based on their scientific background in the area of smart cities from which three were from academia, one policymaker and one consultant engineers), two experts on mobility (one transportation planner and one policymaker), five environmental scientists (three on sustainability, two on strategy development), one expert on economy and one architecture. This collaborative effort ensures a comprehensive and diverse perspective, enhancing the accuracy and depth of the analysis.

The outcomes of the SWOT analysis serve as a valuable foundation for decision-makers, offering strategic insights that can guide effective planning and action in response to the identified factors (D’Adamo et al., 2020). Each expert assessed each methodology based on the following criteria related with the 15-minute concept: (i) proximity, (ii) density, (iii) diversity and (iv) digitalization. Additionally, the fact that within a 15-minute radius, the six essential urban social functions required in all cities should be satisfied: housing, employment, food, health, education and culture – recreation. The experts were questioned after the GIS results and concept of the study were explained while all experts consented in taking part in this analysis and the sharing of their responses. Personal data, apart from occupation, were excluded as per their knowledge. A consent form was also attributed to enhance the safety and handling of the data provided and the results.

## Results and discussion top of form

### GIS results

**Figure 6.**
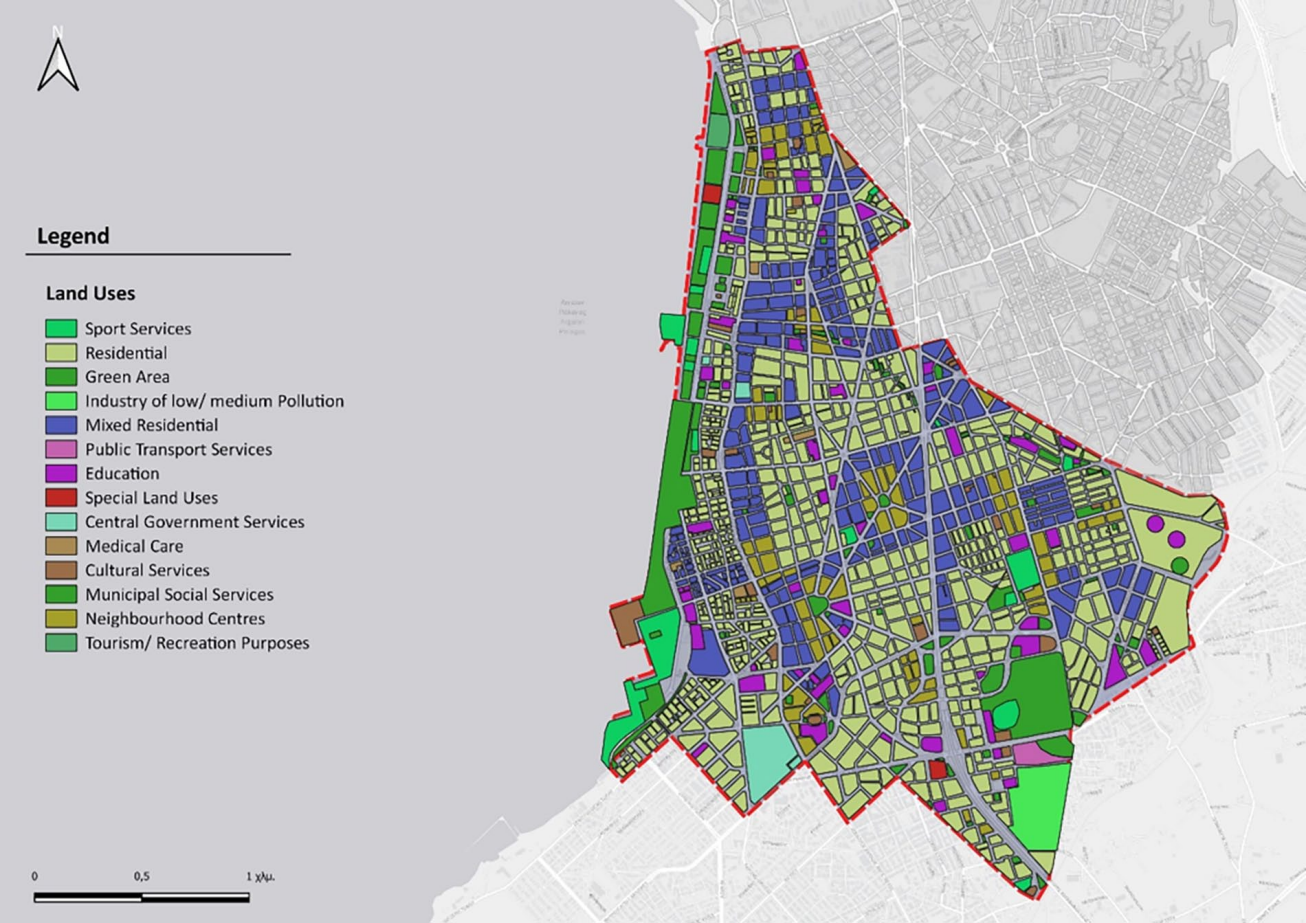
The study area and land uses.

In this section, the results from GIS software for the analysis and evaluation of the ‘15-minute city’ concept in the case of the 5th Municipal Community of Thessaloniki are presented. The analysis was performed for both pedestrian and bicycle movement. Initially, to investigate the accessibility of the study area, the identification of various land uses related to both general and pure residence was a crucial step (Figure 6).

The mapping of the coverage areas according to the respective walking and bicycle use times was based on bibliography. It is generally acknowledged that walking time is a subjective concept, related to both the individual (age, physical condition, etc.) and external conditions (weather, terrain slope, etc.). Most studies assume an average walking speed ranging between 4 and 5 km h^−1^ (Bogusz, 2018; Calafiore et al., 2022; Ferrer-Ortiz et al., 2022) aligning with various other sources, such as the Handbook of Highway Capacity by the Transportation Research Board (2016), recommending a walking speed of 4.8 km h^−1^. Similarly, Google Maps uses an average walking speed of approximately 4.5–5 km h^−1^, which is consistent with common definitions used in related studies (Bohannon and Williams Andrews, 2011; Staricco, 2022). Other studies consider walking speeds lower than 4 km h^−1^, focusing exclusively on specific population groups deemed vulnerable road users, such as children and older adults (Birkenfeld et al., 2023; Bocca, 2021; Willberg et al., 2023). Table 3 presents the radius lengths of the accessibility circles that were used to determine the coverage areas, depending on the type and the time of movement.

**Table 3.** Determination of coverage area according to the type and time of movement.

Movement type	Movement time (min)	Covering area – circle radius (m)
Pedestrian	5	400
	10	800
	15	1200
Bicycle	5	1600
	10	3200
	15	4800

**Figure 7.**
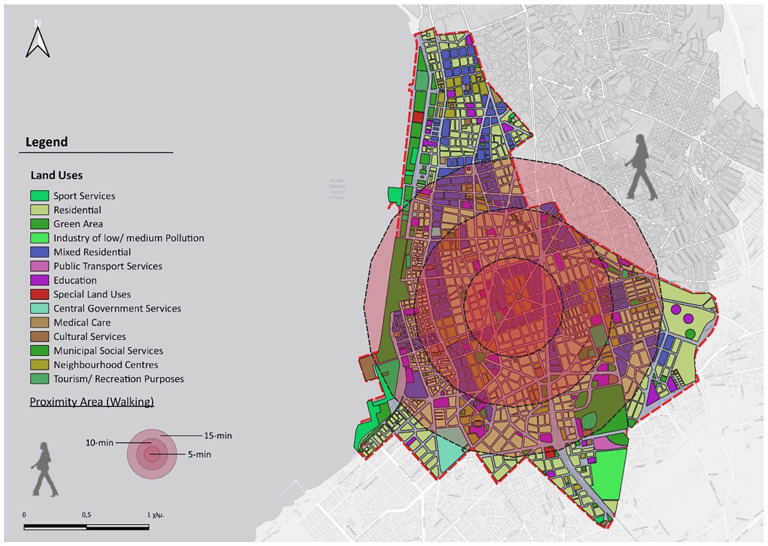
Accessibility circles for walking times of 5, 10 and 15 minutes.

**Figure 8.**
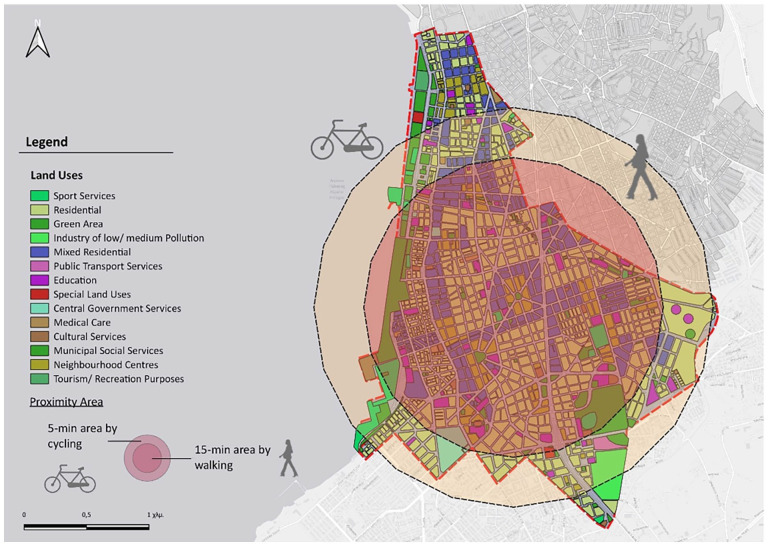
Accessibility circles for a 15-minute walk and a 5-minute bike ride.

Based on the centroid of the 5th Municipal Community, accessibility circles were designed for walking times of 5, 10 and 15 minutes (Figure 7). Respectively Figure 8 shows the accessibility circles for a 15-minute walk and a 5-minute bike ride. It is important to highlight the fact that cycling is assumed and hypothetical, as there are no organized bike lanes throughout the study area, except the one along coastline, incorporated into the paved layout of the beach.

The spatial structure of the study area does not allow for a comprehensive 15-minute mobility plan for all residents, as pedestrian movement leaves some areas at the periphery inaccessible. The region is of mixed use, as almost every building has space for commercial use. Consequently, within these access circles, there is a plethora of various commercial establishments, pharmacies and ATMs. There is also enough cafes and dining places scattered throughout the area. Educational centres are well-distributed within the access circles (15-minute walking and 5-minute cycling). However, some of them are situated at the edges of the study area and cannot be reached within the specified time. The primary challenge at the initial planning level is the lack of knowledge about the number of households requiring education and the specific grade levels.

**Figure 9.**
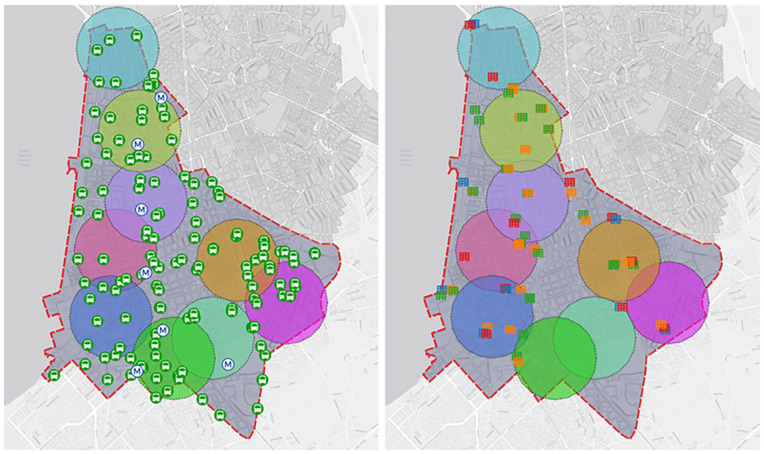
Accessibility circles for 5-minute walking trips. Differences between the levels of proximity for public transport against that of schools can be observed.

Regarding healthcare facilities, there is one private clinic, a small, specialized hospital, and two larger ones, along with a public diagnostic centre within the study boundaries. Additionally, various private examination centres are in different points, although they are not depicted as separate land uses, constituting parts of residential buildings. Determining conclusions for care points is also challenging, as they are limited in number and offer a variety of services to different recipients. Most sports facilities and green spaces are accessible by bicycle, whereas small parks that sometimes include playgrounds, along with parts of the beach, are also reachable by foot. Cultural venues are concentrated along the coastal front, and some are not accessible by walking. The approach with public transportation is very effective in both cases of movement, as the network is dense in the specific area but only in horizontal directions and not transversely (Louro et al., 2019) (Figure 9).

It is important to note that the above results are based on two assumptions. Firstly, all residents of the area walk at the same speed, implying the same travel distance in each time. This is not true, as there are elderly individuals, small children and infants in strollers. Secondly, all movements start from the centre of the area. This is also not accurate in reality, as someone residing at a northern point on the perimeter of the circle will require more time to reach a service at the southeast end of the circle.

### SWOT analysis results

From the analysis of the individual elements of the SWOT, it becomes apparent that the area under study possesses characteristics conducive to a positive transformation into a 15-minute city (Table 4). According to Moreno (2016), the four essential dimensions of planning are adequately met. There is relative diversity in service provision and meeting needs within close distances, supporting pedestrian movement and the population density is conducive to their functionality. In terms of digital infrastructure and digital transformation, the area exhibits a high level, and there is a political inclination towards its further improvement.

**Table 4.** SWOT analysis for15-minute city model’ in study area.

	Strengths	Weaknesses
Internal factors	*Compact urban design*: The study area has a compact urban layout, meaning a high population density that facilitates the implementation of the idea.	*Urban planning and infrastructure*: Narrow streets, dense construction and ageing infrastructure delay the smooth implementation of this idea.
	*Social cohesion*: The study area still maintain a traditional social structure with strong bonds, and citizens have a developed sense of ‘neighbourhood’.	*Lack of open spaces*: There are not enough green spaces or open areas for exercise or community gatherings, with negative implications for health, well-being and socialization of the neighbourhood residents.
	*Mixed land use*: The study area is well-connected with mixed-use neighbourhoods, facilitating the integration of various services and amenities within a 15-minute radius.	*Mindset*: Citizens may not be familiar with sustainable modes of transportation.
	*Existing public transportation network*: The study area has a well-established public transportation network that is easily accessible, contributing to avoiding the use of private cars for commuting to work or other essential needs.	*Funding issues*: Limited resources pose a risk to implementing necessary changes and infrastructure.
	Opportunities	Threats
External factors	*Innovation and technology*: The integration of smart technologies could optimize the effectiveness of this model.	*Climate change*: The increase in temperatures and extreme weather could complicates walking and cycling.
	*European programs*: Possibility of participation in relevant EU programs and financial opportunities.	*International conspiracy theories*: A percentage of citizens in various European countries believe and protest that this design is an attempt by governments to surveil citizens and restrict their freedoms.
	*Tourism development*: It can enhance the attractiveness of study area for tourists, providing them with easily accessible and authentic local experiences, contributing to the industry’s growth.	*Bureaucracy*: Political and regulatory bureaucratic obstacles could potentially slow down the planning and implementation process.

The results of the study of the 15-minute city model indicate that the study area meets the conditions for its implementation based on the four dimensions proposed by Moreno (2016), under certain conditions, as not all needs can be met within these ranges. Especially for work, it is challenging to satisfy even with a 15-minute bicycle travel, as a significant portion of activities is in the western metropolitan area (industrial zone, large hospitals) and in the east (shopping centres, hospitals, airport, research institutes and technology parks). Of course, a considerable number of citizens work in the city centre (commerce, public services, municipality, hotels, catering), considering its significant commercial and business activity (Moreno et al., 2021).

However, the implementation of the design is endangered by inherent problems of the city and its citizens, especially in this specific area. A large number of cars, limited parking spaces, narrow roads, narrow sidewalks, traffic accidents involving pedestrians and cyclists, few green spaces and a bike-unfriendly mentality make it difficult to transition to a different concept of the city. At the same time, according to the US Department of Transportation (2017), to tackle adequate transportation is both vital and complex, hence requiring a multidisciplinary strategy. In 2015, pedestrian and bicyclist fatalities in crashes involving motor vehicles in the United States were reported at 5376 pedestrians and 818 bicyclists while such incidents accounted for a significant 17.7% of all traffic-related deaths that year. Relying solely on isolated measures, whether focused on roadway design improvements or awareness campaigns, falls short of achieving the overarching goal of reducing fatalities and injuries. Instead, a collaborative effort involving various stakeholders is imperative, ranging from governmental leaders and decision-makers to individuals with disabilities and other (Abeliotis et al., 2010; Sharifi, 2020).

Based on the study’s findings, policymakers, urban planner and communities need to focus on several concrete actions to effectively implement the 15-minute city model and advance SDGs. Firstly, they should prioritize promoting sustainable transportation options like walking, cycling and public transit to reduce car dependency, thereby mitigating GHG emissions and promoting healthier lifestyles. Secondly, addressing infrastructure challenges such as heavy car usage, inadequate pedestrian and cyclist infrastructure, and limited green spaces is crucial, necessitating investments in infrastructure upgrades and green space expansion. Thirdly, policymakers should adopt a multidisciplinary approach and collaborate with various stakeholders to address challenges comprehensively, ensuring coordination between transportation agencies, urban planners and community groups. Overall, policymakers must adopt integrated approaches that address both physical infrastructure and social dynamics to achieve genuine and widespread engagement in sustainable urban initiatives (Bonello et al., 2022; Hu et al., 2023).

Even though informing residents about behaviour change and involving them in all stages of planning is a crucial part of the inclusive and equitable approach proposed by the 15-minute city model, infrastructure works and interventions are needed to convince citizens of the health benefits, both physical and mental (Biancardi et al., 2023a). These actions may include sidewalk improvements, development of a bike lane network, establishment of parking spaces, creation of bike-sharing system installation areas, smart crosswalks, tree planting and restructuring of the public transportation system, among others (Moreno et al., 2021).

In general, however, for a successful implementation of any strategy, urban planning initiative, strategy and legislation, social acceptance, willing participation and education of sustainable development is crucial (Biancardi et al., 2023b). This vitality for social acceptance as well as addressing the social aspect of urban planning and development has indeed captured the attention of the scientific community over the past few decades (Di Fiore et al., 2022; Eliades et al., 2022; Kwon and Silva, 2019; Mensah, 2021; Voukkali et al., 2023a). This is directly reflected by the results of the SWOT analysis, as one of the weaknesses identified by the experts was the change in the mindset of citizens who might not be familiar with the alteration towards sustainable transportation. At the same time, one of the main threats is the existence of ‘international conspiracies’ where a large portion of European citizens that smart, green or 15-minute city designs is performed in order for governments to surveil the population more closely (Biesaga et al., 2023).

Hence, the willing and active participation of citizens in the 15-minute approach implementation can be achieved through the psychological understanding of the factors that affect their participation (Ardoin et al., 2020). According to research (Fan et al., 2020; Kaufmann et al., 1998; Scott et al., 2023; Zorpas et al., 2017), higher income levels are generally associated with greater willingness of citizens to participate in sustainable initiatives. Still, this correlation is heavily influenced by other factors like environmental education, social attitude, cultural background which are somewhat ‘personalized’ and customized to the area under investigation (Papamichael et al., 2022).

The implementation of ‘15-minute city approach’ is closely aligned with the broader goals of sustainable urban development (Khavarian-Garmsir et al., 2023; Logan et al., 2022). The study explores the feasibility of implementing the 15-minute city model, which emphasizes localized living and reduced reliance on cars, thereby contributing to the mitigation of climate change. By promoting sustainable transportation options, the research aligns with efforts to create more environmentally friendly and resilient cities. Moreover, the study provides valuable insights for urban planners to design and implement infrastructure improvements that enhance the liveability and sustainability of cities. Furthermore, the emphasis on public engagement and community involvement underscores the significance of social sustainability in urban development. By involving residents in the planning process and considering their needs and preferences, the research advocates for inclusive and equitable urban policies that enhance social cohesion and equity. This corresponds with the wider objective of building cities that are accessible, inclusive and attentive to the needs of all residents.

Concluding, by delving into the specific conditions and requirements identified within the study area, cities gain invaluable insights into the intricacies of implementing the 15-minute city model. This understanding enables them to adapt and customize their strategies and interventions to better suit the unique characteristics of their own urban environments. For example, if a city faces similar challenges, it can draw upon the approaches and solutions identified in the research to develop personalized initiatives that address these specific issues. Additionally, the results offer a repository of valuable lessons and best practices that transcend geographical boundaries and can be applied to diverse urban settings. These insights serve as a roadmap for cities embarking on their journey towards implementing the 15-minute city concept – whether its leveraging community engagement strategies, prioritizing infrastructure improvements or adopting interdisciplinary approaches.

## Conclusion

In an era where cities hold increased economic and political influence, utilizing advanced technology to enhance their activities, they simultaneously encounter substantial sustainability challenges across various interconnected systems. The industrial revolution has had a profound impact on urban environments, pose significant issues that limit individual and collective well-being.

Ensuring planetary sustainability has become a prominent urban challenge, requiring cities to reduce GHGs emissions, strengthen resilience to environmental changes, improve residents’ health, reshape economies and address growing inequalities. Recognizing the need for transformative changes, various design models have been proposed and evolved based on diverse implementation experiences. The ‘15-minute city’ has emerged as a novel urban model, envisioning a polycentric yet human-centric city, with time as a central design element. By reducing reliance on cars, this model aims to minimized emissions, foster cleaner environments and mitigate climate change impacts.

However, the applicability of the ‘15-minute city’ is contingent on the unique characteristics of each city’s urban and social structures, legislative provisions and governance. Although European cities, including Greek cities, are seen as inherently suited for this model, successful implementation requires infrastructure projects supported by political will, secure funding and citizen acceptance. Gradual implementation, starting with small interventions, is recommended for success, and complementary policies are essential to encourage active and sustainable mobility.

The study’s findings underscore the urgency for urban planners and policymakers to embrace transformative changes in response to the sustainability challenges faced by cities. In light of the substantial impact of the industrial revolution on urban environments and the pressing need to address issues limiting well-being, there is a clear imperative to strengthen the call to action. The emergence of innovative urban models like the ‘15-minute city’ offers a promising avenue for addressing these challenges by prioritizing human-centric design and reducing reliance on cars to minimize emissions and mitigate climate change impacts. However, the successful implementation of such models hinges on factors such as urban and social structures, legislative provisions, governance, political will, funding and citizen acceptance.

Finally, the main limitations identified in this study, which can be addressed in future research, include as mentioned above the methodological rigour. More specifically, the choice of the Euclidean distance for the circular buffers was preferred against the road network buffers (i.e. isochrones) as the last method due to complexity and the need for a higher level of computer resources. Therefore, the level of accuracy regarding proximity analysis can be enhanced in a more detailed analysis. Moreover, existing origin–destination trips were not included in the analysis. The ability to understand the distribution of the existing trips in the current transport infrastructure can enhance the analysis for implementing the 15-minute city concept in the study area. Finally, social opinion for the implication of the 15-minute city in the examined study area can highlight the level of acceptability of this concept.
